# Supplementary material for: Pet Reptiles—Are We Meeting Their Needs?
Source: Animals (Basel). 2021 Oct 14;11(10):2964. doi: 10.3390/ani11102964 (PMC8533019; doi:10.3390/ani11102964)
Supplement: Supplementary file 1 [file animals-11-02964-s001.zip › Supplementary materials/Table S1. Questionnaire for chelonians, snakes and lizards (Translated).pdf]

**Table S1.** Full questionnaires for chelonians, snakes and lizards.

## Questionnaire

This Questionnaire is intended for reptile keepers, particularly chelonians / snakes / lizards and is part of a larger survey.

The aim of the study is to analyze the keeper-reptile bond and to understand its effects on reptile welfare, in Portugal.

The participation is voluntary and its completion takes an average of 15 minutes. The collected data will be used for scientific purposes and will not be shared with third parties. The identity of the participants will not be revealed.

If you wish to know more about this study, you can contact the responsible researcher, Leonor Guimarães, using the following email: mleonor93@gmail.com.

### Part 1 – General questions about the animal

Note: If you have more than one chelonian/lizard/snake, consider only one (always the same one) to answer the questions.

| | |
|---|---|
| What are the scientific and common names of the species? | |
| Birth date | Born in ______(year) or<br>Before ________(year)<br>After ______ (year) |
| How long ago did you acquire it? | |
| Sex | Male ☐ Female ☐ Don't know ☐ |
| Is it your first reptile pet? | Yes ☐ No ☐ |
| Where did you acquire it? | Pet store ☐<br>Offered (gift) ☐<br>Captured in the wild ☐<br>Other (please specify) ☐<br>________________________ |

| | |
|---|---|
| Do you have other pets in your house, at the moment? Which ones? | |
| Did you have other pets in the past? Which ones? Ex: I had dogs, cats, budgerigars | |

**Part 2 - Husbandry Questions**

**Definitions**

***Terrarium:** enclosure where the environmental conditions needed for maintaining terrestrial and semi-terrestrial species are artificially reproduced (e.g. humidity, lighting and temperature).

***Aquarium:** enclosure where the environmental conditions needed for maintaining aquatic or semi-aquatic species are artificially reproduced.

***Cage:** an enclosure often made of mesh, bars, or wire fencing.

| | |
|---|---|
| Environment | Indoor ☐ Outdoor ☐ Both ☐ |
| Housing<br>(choose all aplicable) | Terrarium* ☐ Aquarium* ☐<br>Cage *☐ Free roaming ☐<br>Other ☐ ______________________ |
| Please specify enclosure dimensions | (length) _____cm x (width) _____cm x (height) _____cm |
| How many animals share the enclosure? | |
| Which other animals share the enclosure with the chelonian/snake/lizard?<br>(please specify) | Other reptile of the same group ☐<br>Other reptile of a different group ☐<br>Housed alone ☐<br>Other ☐ ______________________ |

| | |
|---|---|
| The enclosure has: | Transparent walls (glass) ☐<br>Reflective walls (mirror-like) ☐<br>Opaque walls ☐<br>Several of the above☐ |
| Does the reptile have access to a source of sunlight or UVB light?<br>(choose all applicable) | Yes, sunlight through the window glass ☐<br>Yes, direct sunlight ☐<br>Yes, UVB lamp outside the enclosure ☐<br>Yes, UVB lamp within the enclosure ☐<br>No ☐ Don't know ☐ |
| Do you provide a light-dark cicle? | Yes ☐ No ☐ Don't know ☐ |
| The enclosure has:<br>(choose all applicable) | Heat source ☐ Heat lamp ☐<br>UVB lamp ☐ Fluorescent Lamp ☐<br>Lamp but I don´t know the type ☐<br>None of the above☐<br>Other ☐ ______________________ |
| What kind of heat source do you provide? | |
| Please specify the temperatures of the enclosures:<br>- maximum (hot spot)<br>- minimum (cool end)<br>- ambient (average) | |
| Are there hiding spaces? | Yes ☐ No ☐ Don't know ☐ |
| In the case of being a semi-aquatic turtle, does it have access to dry areas or platforms? | Yes ☐ No ☐ Don't know ☐ |

| | |
|---|---|
| What kind of substrate is there on the enclosure?<br><br>(choose all applicable) | Water (enough for the reptile to swim) ☐<br>Shallow water (enough for bathing) ☐<br><br>Sand ☐ Wood shavings ☐ Bark shavings ☐<br><br>Egg-laying substrate ☐<br><br>Moss ☐ Humus ☐<br><br>Clay ☐ Soil ☐<br><br>Other ☐ ________________ |
| Can you describe the frequency of cage cleaning? | |
| Feeding (chelonians)<br><br>(choose all applicable) | Commercial feed (pellets) ☐ Fruit ☐ Vegetables ☐ Insects ☐<br><br>Dried shrimp ☐ Fish ☐ worms ☐<br><br>Other ☐_____________ |
| Feeding (snakes)<br><br>(choose all applicable) | |
| Feeding items (lizards)<br><br>(choose all applicable) | |
| Do you provide vitamin or mineral supplements? Can you describe which ones and how you apply them? (ex: vitamin A, vitamin D, calcium on the feed) | |

**Part 3 - Reptile-owner relationship**

**Definitions**

***Welfare**: a state in which the animal is free of hunger, thirst, discomfort, pain, injury, disease and fear, and has the freedom to express its normal behaviour**.**

| | |
|---|---|
| Which definition best describes your reptile?<br>(choose all applicable) | It's a family member ☐ It's a friend ☐<br>It's a pet ☐ It's a burden ☐<br>I have no opinion ☐<br>Other ☐ ______________________ |
| Do you have the habit of talking with your reptile? | No ☐ Yes ☐<br>If yes, how many times a week?<br>1 ☐ 2 ☐ 3 ☐ 4 ☐ 5 ☐ >5 ☐ |
| Do you have the habit of petting your reptile? | No ☐ Yes ☐<br>If yes, how many times a week?<br>1 ☐ 2 ☐ 3 ☐ 4 ☐ 5 ☐ >5 ☐ |
| Do you have the habit of handling your reptile? (e.g. putting it on your lap our transporting it) | No ☐ Yes ☐<br>If yes, how many times a week?<br>1 ☐ 2 ☐ 3 ☐ 4 ☐ 5 ☐ >5 ☐ |
| When you approach your reptile, what reactions do you observe most frequently? (choose all applicable) | No reaction ☐ Approaches me ☐<br>Head lifting ☐<br>Moves away (tries to escape) ☐<br>Hiding ☐<br>Retraction into shell (chelonians) ☐<br>Digging/Burrowing ☐ Vocalizes ☐<br>Mouth opening ☐<br>Biting ☐ Other☐ _____________ |
| How would you rate the experience of keeping a reptile pet, in general terms? | Very good ☐ Good ☐<br>Bad ☐ Very bad ☐<br>I have no opinion ☐ |
| Since you acquired your reptile, how many times did you take it to a veterinary consultation? | Never ☐<br>1 ☐ 2 ☐ 3 ☐ 4 ☐ 5 ☐ >5 ☐ |
| The veterinary consultations are for:<br>(choose all applicable) | Routine/ General Check-up ☐<br>When I notice a change ☐<br>Other ☐_____________ |

| | |
|---|---|
| In your opinion, how would you classify the *welfare of your reptile pet?<br>(please choose a number from 1 to 5, with 1 being very bad and 5 very good) | 1☐ 2 ☐ 3 ☐ 4 ☐ 5 ☐ |

**Part 4 - Behaviour Questions:**

Please choose, in your opinion, the causes associated with the following behaviours. For each sentence, choose all of the answers you consider appropriate

**Definitions**

***Normal behaviour:** a behaviour that is natural for the species and related to good welfare

***Welfare:** a state in which the animal is free of hunger, thirst, discomfort, pain, injury, disease and fear, and has the freedom to express its natural behaviour.

| **Behaviour** | **Possible Causes** |
|---|---|
| The reptile moves around in the enclosure investigating objects /people and exploring the environment. | Normal behaviour ☐<br>Communication ☐ Stress/fear ☐ Escape behaviour ☐ Pain ☐ Dysfunction/disease ☐ Food searching ☐ Reproductive behaviour ☐ Hibernation ☐<br>Hot ☐ Cold ☐ Other: _______________ |
| The reptile basks under the sunlight, UVB lamp or heat source with extended limbs and head. | Normal behaviour ☐<br>Communication ☐ Stress/fear ☐ Escape behaviour ☐ Pain ☐ Dysfunction/disease ☐ Food searching ☐ Reproductive behaviour ☐ Hibernation ☐<br>Hot ☐ Cold ☐ Other: _______________ |
| Persistent (very frequent) attempts | Normal behaviour ☐ |

| | |
|---|---|
| to push against, crawl up, dig under or round the enclosure barriers. | Communication ☐ Stress/fear ☐ Escape behaviour ☐ Pain ☐ Dysfunction/disease ☐ Food searching ☐ Reproductive behaviour ☐ Hibernation ☐<br>Hot ☐ Cold ☐ Other: ________________ |
| Decreases in activity and/or appetite. | Normal behaviour ☐<br>Communication ☐<br>Stress/fear ☐ Escape behaviour ☐ Pain ☐ Dysfunction/disease ☐ Food searching ☐ Reproductive behaviour ☐ Hibernation ☐<br>Hot ☐ Cold ☐ Other: ________________ |
| Human-directed aggression, e.g. biting or striking. | Normal behaviour ☐<br>Communication ☐ Stress/fear ☐ Escape behaviour ☐ Pain ☐ Dysfunction/disease ☐ Food searching ☐ Reproductive behaviour ☐ Hibernation ☐<br>Hot ☐ Cold ☐ Other: ________________ |
| Retraction of head, limbs or tail into the shell in response to human presence or manipulation (chelonians). | Normal behaviour ☐<br>Communication ☐ Stress/fear ☐ Escape behaviour ☐ Pain ☐ Dysfunction/disease ☐ Food searching ☐ Reproductive behaviour ☐ Hibernation ☐<br>Hot ☐ Cold ☐ Other: ________________ |
| Open-mouth breathing with extended neck. | Normal behaviour ☐<br>Communication ☐ Stress/fear ☐ Escape behaviour ☐ Pain ☐ Dysfunction/disease ☐ Food searching ☐ Reproductive behaviour ☐ Hibernation ☐<br>Hot ☐ Cold ☐ Other: ________________ |
| Cloacal discharge (faeces or urine) | Normal behaviour ☐ |

| | |
|---|---|
| or regurgitation in response to human presence or manipulation | Communication ☐ Stress/fear ☐ Escape behaviour ☐ Pain ☐<br>Dysfunction/disease ☐ Food searching ☐<br>Reproductive behaviour ☐ Hibernation ☐<br>Hot ☐ Cold ☐ Other: ________________ |
| Occupying a dark area, refuge or hiding box. | Normal behaviour ☐ |
| | Communication ☐ Stress/fear ☐<br>Escape behaviour ☐ Pain ☐<br>Dysfunction/disease ☐ Food searching ☐<br>Reproductive behaviour ☐ Hibernation ☐<br>Hot ☐ Cold ☐ Other: ________________ |

**Part 5 – Owner Information**

| | |
|---|---|
| What type of owner are you? | Private ☐ Breeder ☐ |
| Age | |
| Gender | |
| District | |
| Environment where you live | Urban ☐ Rural ☐ |
| Education level | Elementary School ☐<br>Middle School ☐<br>High School ☐<br>Graduation ☐<br>Master's degree ☐<br>Doctorate ☐ |
| Profession | |
| If you are a student please choose your field of study | Art ☐ Science ☐<br>Humanities ☐ Economics ☐<br>Other:________________________ |

| | |
|---|---|
| Marital Status | |
| Household | Apartment ☐ House ☐ |
| Garden | Yes ☐ No ☐ |

**Part 6 – Optional Question**

| | |
|---|---|
| Please explain, in a few words, why did you decide to acquire and keep a reptile pet | |

**The End. Thank you for your participation**
