# Supplementary material for: Pet Reptiles—Are We Meeting Their Needs?
Source: Animals (Basel). 2021 Oct 14;11(10):2964. doi: 10.3390/ani11102964 (PMC8533019; doi:10.3390/ani11102964)
Supplement: Supplementary file 1 [file animals-11-02964-s001.zip › Supplementary materials/Table S2. Data preparation.pdf]

**Table S2** – Data preparation

**A.** List of variables collected from survey and rationale for inclusion in model building. The decision or not to include variables in modelling was based on literature review and consensus among the authors regarding the relevance in daily clinical practice (e.g. importance of temperature for the incidence of subclinical hypothermia or thermal injury, or of interaction with boundaries for rostral abrasion).

| **Snakes - section 2 - husbandry** | | |
|---|---|---|
| **Survey question** | **Variable** | **Justification** |
| Environment (indoor, outdoor, both) | not selected for inferential analysis | Insufficient information to assess adequacy. All snakes were kept in indoor or in mixed scenarios. |
| Enclosure type (terrarium, aquarium, cage, free-roaming, other) | not selected for inferential analysis | Insufficient information to assess adequacy. Whether the enclosure type meets the needs of the reptile depends on many factors such as species, enclosure design, animal size, heat and light sources, etc. |
| Enclosure dimension (length x width x height) | not selected for inferential analysis | Insufficient information to assess adequacy. Adequacy of enclosure dimension is highly dependent on specimen size, species, enclosure design, and other husbandry variables. |
| Co-habitants | not selected for inferential analysis | All snakes were housed alone or with up to 3 specimens of the same species. As most are solitary species but tolerate conspecifics, this variable was not predicted to be a relevant for inferential analysis |

| | | |
|---|---|---|
| Enclosure walls (transparent, opaque, mixed) | not selected for inferential analysis | Opaque enclosure walls could reduce stress by acting as visual barriers against humans and other aversive stimuli; transparent walls are considered positive enrichment in species that exhibit vigilance behaviour such as cats; it stands logical that mixed set-ups with opaque and transparent sections could allow the best of both options by enabling behavioural self-regulation and choice. Totally transparent enclosure walls were considered inadequate, mixed and opaque walls were considered adequate. Nevertheless, due to the variability in species responses to human presence and dependence on enclosure size and setup, there was insufficient information to assess adequacy. |
| Access to light | no access = inadequate; access = adequate | In the case of carnivorous reptiles like snakes, it is unclear whether UV light is crucial for vitamin D sufficiency; however, while possessing pituitary glands and photoperiodic regulation of life-history events, no access to light was considered inadequate for the purpose of this study. |
| Hours of darkness | not selected for inferential analysis | All reptiles had access to hours of darkness. |
| Enclosure equipment (heat source, heat lamp, UVB lamp, unknown lamp, lamp, other) | not selected for inferential analysis | Insufficient information to assess adequacy. Depends heavily on species and enclosure set-up. Having no heat source or no light source would be considered inadequate, but is accounted for in variables temperature and UV light. |
| Type of heat source (mat, lamp, cord, plate, inside vs. outside) | not selected for inferential analysis | Insufficient information to assess adequacy. Depends heavily on species and enclosure set-up. Having no heat source or no light source would be considered inadequate, but is accounted for in variables temperature and UV light. |

| | | |
|---|---|---|
| Temperature range (min-max) | High-end temperatures more than 2ºC below or 5ºC or more above the reference = inadequate; high-end temperature with -2 to +4ºC of the reference = adequate. | The adequacy of the temperature range is dependent on species, population within species (e.g. differences in montane or tropical populations) and size. Nevertheless, indicative values are available for many species based on the environmental conditions in the wild and empirical experience in captive settings. High-end temperatures more than 2ºC below or 5ºC or more higher than the reference were considered inadequate. |
| Relative humidity (% value) | not selected for inferential analysis | Insufficient information to assess adequacy. The adequacy of relative humidity is dependent on species, population within species (e.g. differences in montane or tropical populations), life-stage and size. |
| Tri-dimensional enrichment with logs or rocks | not selected for inferential analysis | Most reported species are semi-arboreal (e.g. ball python); even terrestrial species may use vertical structures; the absence of tri-dimensional enrichment structures was considered inadequate. However, there was no uniform criterion across taxonomic groups that would allow inclusion into the models. |
| Refuges or hiding places | absent = inadequate; present= adequate | All snakes can exhibit hiding behaviour to some extent, relying on camouflage and immobility to avoid predation; the absence of places to hide or take refuge was considered inadequate because it impedes the expression of behaviours that have high survival value and probably high internal motivation. |
| Substrate (swimming water, drinking water, moss, bark, wood shavings, clay, coconut fibre, kitchen paper, etc...) | not selected for inferential analysis | Several substrates are used for keeping snakes and their adequacy is context-dependent. For example, where sanitary conditions are the main concern, kitchen paper can be highly effective, but for the expression of burrowing behaviour it could be inadequate. For an arboreal species, the choice between both could be irrelevant. It was not possible to create a uniform criterion for all species. |

| | | |
|---|---|---|
| Frequency of enclosure higiene | not selected for inferential analysis | context dependent, descriptive analysis only |
| Feeding | not selected for inferential analysis | context dependent, descriptive analysis only |
| Feeding frequency | not selected for inferential analysis | context dependent, descriptive analysis only |
| Suplements (vitamin D, calcium, none) | not selected for inferential analysis | Not considered extremely relevant for carnivorous snakes on a whole prey diet. |
| Diet | whole prey diet = adequate; other=inadequate | A diet based on whole prey items was considered adequate for snakes. The feeding of live prey could be considered inadequate in many cases, but this is context dependent, and there was insufficient information to assess it. |
| **Snakes - section 3 - bond** | | |
| **Survey question** | **Variable** | **Justification** |
| What better defines your snake (family-member, friend, pet, burden, other) | family member = 1 vs. other bond = 0 | For use as a dependant variable, bond was transformed into a binomial variable to allow logistic regression analysis. Viewing the reptile as a family member was considered the closest bond. |
| How often do you speak to your reptile per week? (never, 1, 2, 3, 4, 5, >5 time) | categorical: never, 1-5 times per week, daily | The responses were collapsed into 3 categories to maintain degrees of freedom manageable. |
| How often do you pet your reptile per week? (never, 1, 2, 3, 4, 5, >5 time) | categorical: never, 1-5 times per week, daily | The responses were collapsed into 3 categories to maintain degrees of freedom manageable. |

| | | |
|---|---|---|
| How often do you handle your reptile per week, e.g. picking it up or putting it in your lap? (never, 1, 2, 3, 4, 5, >5 time) | categorical: never, 1-5 times per week, daily | The responses were collapsed into 3 categories to maintain degrees of freedom manageable. |
| When you approach your reptile, how does it react? (approaches, moves away, lifts head, hides, opens mouth, digs, bites, no reaction) | not selected for inferential analysis | For use as an independent variable, behaviours were grouped as predominantly indicative of positive states (e.g. approaching), negative states (biting, evading, hiding, hissing, puffing up) or indeterminate (no reaction). Many responses were difficult to accurately classify, and therefore this variable was not chosen for inferential analysis. |
| How would you rate the experience of keeping a reptile pet? (very good, good, bad, very bad, no opinion) | binomial: very good or good vs. other response | Keeping reptiles might be tiresome and scarce in gratifying experiences, resulting in a mostly unilateral relation. This could reflect the level of care. |
| How many times have you taken your pet to the veterinarian since you have it? (1, 2, 3, 4, 5, >5) | continuous variable; not selected for inferential analysis. | Divided the number of times reported by the number of years kept as pet. Might need to consider log transformation due to the leverage of frequent visits in very young pets causing outlier effects. |
| Cause of visits to veterinary practice (routine, illness, other) | binomial: routine vs. illness or never | Reptiles commonly mask signs of illness to avoid predation, and therefore are often in life-threatening condition once they start to present clinical signs. Therefore, routine veterinary care, even if infrequent, is considered necessary to ensure reptile welfare. Further, routine veterinary care could provide a source for information on behaviour, reptile care and husbandry. |

| | | |
|---|---|---|
| How would you rate you reptile's welfare (very poor, poor, average, good, very good), considering the following definition: *the animal is free of hunger, thirst, discomfort, pain, lesion, illness and fear, and is able to express its normal behaviour.* | binomial: good or very good vs. average or less. | Based on the distribution of the answers, the variable was coded as a binomial variable. |
| **Snakes - section 4 - behaviour** | | |
| **Survey question** | **Variable** | **Justification** (adapted from Warwick et al. 2013) |
| The reptile investigates the environment, approaching and smelling objects and/or people | normal behaviour = adequate | Was considered to be related to the expression of exploratory and foraging behaviour. |
| The reptile exposes itself to light (sunlight or lamp) or approaches the heat source | normal behaviour = adequate | Thermoregulatory behaviour of ectotherms; inability to express this behaviour is likely to be associated with poor welfare. |
| The reptile repeatedly hits its head on the glass or enclosure wall and/or tries to climb the enclosure wall | normal behaviour = inadequate | Head-bumping or abrading the rostral region on enclosure walls is associated with captivity stress and interaction with transparent boundaries; could be related with the inability to express a behaviour for which motivation exists such as exploration, thermoregulation, foraging. |
| Decreased physical activity and/or appetite | normal behaviour = inadequate | Decreased physical activity and appetite compared to normal levels are associated with hypothermia, pathology, discomfort/pain, weakness |
| Exhibits aggressive behaviour towards humans (e.g. tries to bite) | normal behaviour = inadequate | Aggressive behaviour toward humans in reptiles can be defensive, and thus associated with fear. |

| | | |
|---|---|---|
| Fast open-mouth breathing with extended neck | normal behaviour = inadequate | Fast open-mouth breathing and gasping for air with extended neck are associated with dyspnoea or hyperthermia |
| The reptile urinates/defecates or regurgitates when in contact or presence of human | normal behaviour = inadequate | Cloacal discharges and regurgitation are defensive behaviours of reptiles and are triggered by fear or stress responses. |
| The snake has difficulty in coiling its body | not selected for inferential analysis | difficulty in coiling is associated with pain, weakness or illness |
| The reptile moves to a dark place in the enclosure or refuge | not selected for inferential analysis | Ambiguous. Could be related to both positive and negative states, for example brumation, cryptic behaviour, and thermoregulation. |
| The reptile spends long periods in water | not selected for inferential analysis | Ambiguous. Could be related to both positive and negative states, for example defecation/urination, thermoregulation, ecdysis. |

**Lizards - section 2 - husbandry**

| **Survey question** | **Variable** | **Justification** |
|---|---|---|
| Environment (indoor, outdoor, both) | not selected for inferential analysis | Insufficient information to assess adequacy. All lizards were kept in indoor or in mixed scenarios. |
| Enclosure type (terrarium, aquarium, cage, free-roaming, other) | not selected for inferential analysis | Insufficient information to assess adequacy. Whether the enclosure type meets the needs of the reptile depends on many factors such as species, enclosure design, animal size, heat and light sources, etc. |
| Enclosure dimension (length x width x height) | not selected for inferential analysis | Insufficient information to assess adequacy. Adequacy of enclosure dimension is highly dependent on specimen size, species, enclosure design, and other husbandry variables. |

| | | |
|---|---|---|
| Co-habitants | not selected for inferential analysis | All lizards were housed alone or with up to 3 specimens of the same species. As most are solitary species but tolerate conspecifics, this variable was not predicted to be a relevant for inferential analysis |
| Enclosure walls (transparent, opaque, mixed) | not selected for inferential analysis | Opaque enclosure walls could reduce stress by acting as visual barriers against humans and other aversive stimuli; transparent walls are considered positive enrichment in species that exhibit vigilance behaviour such as cats; it stands logical that mixed set-ups with opaque and transparent sections could allow the best of both options by enabling behavioural self-regulation and choice. Totally transparent enclosure walls were considered inadequate, mixed and opaque walls were considered adequate. Nevertheless, due to the variability in species responses to human presence and dependence on enclosure size and setup, there was insufficient information to assess adequacy. |
| Access to unfiltered UVB light (sunlight or lamp) | no access = inadequate; access = adequate | For omnivorous and herbivorous lizards UV or direct sunlight are crucial for survival and welfare. Failure to provide this resource results in MBD, a condition accompanied by pain, muscle weakness, fractures, etc. |
| Hours of darkness | not selected for inferential analysis | All reptiles had access to hours of darkness. |
| Enclosure equipment (heat source, heat lamp, UVB lamp, unknown lamp, lamp, other) | not selected for inferential analysis | Insufficient information to assess adequacy. Depends heavily on species and enclosure set-up. Having no heat source or no light source would be considered inadequate, but is accounted for in variables temperature and UV light. |

| | | |
|---|---|---|
| Type of heat source (mat, lamp, cord, plate, inside vs. outside) | not selected for inferential analysis | Insufficient information to assess adequacy. Depends heavily on species and enclosure set-up. Having no heat source or no light source would be considered inadequate, but is accounted for in variables temperature and UV light. |
| Temperature range (min-max) | High-end temperatures more than 2ºC below or 5ºC or more above than the reference = inadequate; high-end temperature with -2 to +4ºC of the reference = adequate. | The adequacy of the temperature range is dependent on species, population within species (e.g. differences in montane or tropical populations) and size. Nevertheless, indicative values are available for many species based on the environmental conditions in the wild and empirical experience in captive settings. High-end temperatures more than 2ºC below or 5ºC or more higher than the reference were considered inadequate. |
| Relative humidity (% value) | not selected for inferential analysis | Insufficient information to assess adequacy. The adequacy of relative humidity is dependent on species, population within species (e.g. differences in montane or tropical populations), life-stage and size. |
| Tri-dimensional enrichment with logs or rocks | not selected for inferential analysis | Even terrestrial species may use vertical structures; the absence of tri-dimensional enrichment structures was considered inadequate. However, there was no uniform criterion across taxonomic groups that would allow inclusion into the models. |
| Refuges or hiding places | absent = inadequate; present= adequate | The absence of places to hide or take refuge was considered inadequate because it impedes the expression of behaviours that have high survival value and probably high internal motivation. |

| | | |
|---|---|---|
| Substrate (swimming water, drinking water, moss, bark, wood shavings, clay, coconut fibre, kitchen paper, etc...) | not selected for inferential analysis | Several substrates are used for keeping lizards and their adequacy is context-dependent. For example, where sanitary conditions are the main concern, kitchen paper can be highly effective, but for the expression of burrowing behaviour it could be inadequate. For an arboreal species, the choice between both could be irrelevant. It was not possible to create a uniform criterion for all species. |
| Frequency of enclosure hygiene | not selected for inferential analysis | context dependent, descriptive analysis only |
| Feeding (live vs. dead; invertebrates, mammals, birds, vegetables, fruit, etc…) | Ca and vit. D provided in some form = adequate; not provided = inadequate | Considered in combination. Relevant for lizards in captivity. Inadequate: absence of dietary source of vitamin D3 and Ca. |
| What kind, frequency and method is used for supplementation (vit D3, Ca, multivit, in formulated feed, dusted, mixed with food, etc…) | | |
| **Lizards - section 3 - bond** | | |
| **Survey question** | **Variable** | **Justification** |
| What better defines your lizard (family-member, friend, pet, burden, other) | family member = 1 vs. other bond = 0 | For use as a dependant variable, bond was transformed into a binomial variable to allow logistic regression analysis. Viewing the reptile as a family member was considered the closest bond. |
| How often do you speak to your reptile per week? (never, 1, 2, 3, 4, 5, >5 time) | categorical: never, 1-5 times per week, daily | The responses were collapsed into 3 categories to maintain degrees of freedom manageable. |

| | | |
|---|---|---|
| How often do you pet your reptile per week? (never, 1, 2, 3, 4, 5, >5 time) | categorical: never, 1-5 times per week, daily | The responses were collapsed into 3 categories to maintain degrees of freedom manageable. |
| How often do you handle your reptile per week, e.g. picking it up or putting it in your lap? (never, 1, 2, 3, 4, 5, >5 time) | categorical: never, 1-5 times per week, daily | The responses were collapsed into 3 categories to maintain degrees of freedom manageable. |
| When you approach your reptile, how does it react? (approaches, moves away, lifts head, hides, opens mouth, digs, bites, no reaction) | not selected for inferential analysis | For use as an independent variable, behaviours were grouped as predominantly indicative of positive states (e.g. approaching), negative states (biting, evading, hiding, hissing, puffing up) or indeterminate (no reaction). Many responses were difficult to accurately classify, and therefore this variable was not chosen for inferential analysis. |
| How would you rate the experience of keeping a reptile pet? (very good, good, bad, very bad, no opinion) | binomial: very good or good vs. other response | Keeping reptiles might be tiresome and scarce in gratifying experiences, resulting in a mostly unilateral relation. This could reflect the level of care. |
| How many times have you taken your pet to the veterinarian since you have it? (1, 2, 3, 4, 5, >5) | continuous variable; not selected for inferential analysis. | Divided the number of times reported by the number of years kept as pet. Might need to consider log transformation due to the leverage of frequent visits in very young pets causing outlier effects. |
| Cause of visits to veterinary practice (routine, illness, other) | binomial: routine vs. illness or never | Reptiles commonly mask signs of illness to avoid predation, and therefore are often in life-threatening condition once they start to present clinical signs. Therefore, routine veterinary care, even if infrequent, is considered necessary to ensure reptile welfare. Further, routine veterinary care could provide a source for information on behaviour, reptile care and husbandry. |

| | | |
|---|---|---|
| How would you rate you reptile's welfare (very poor, poor, average, good, very good), considering the following definition: *the animal is free of hunger, thirst, discomfort, pain, lesion, illness and fear, and is able to express its normal behaviour.* | binomial: good or very good vs. average or less. | Based on the distribution of the answers, the variable was coded as a binomial variable. |
| **Lizards - section 4 - behaviour** | | |
| **Survey question** | **Variable** | **Justification** (adapted from Warwick et al. 2013) |
| The reptile investigates the environment, approaching and smelling objects and/or people | normal behaviour = adequate | Was considered to be related to the expression of exploratory and foraging behaviour. |
| The reptile exposes itself to light (sunlight or lamp) or approaches the heat source | normal behaviour = adequate | Thermoregulatory behaviour of ectotherms; inability to express this behaviour is likely to be associated with poor welfare. |
| The reptile frequently bumps its head on the glass or enclosure wall or tries to climb the enclosure wall | normal behaviour = inadequate | Head-bumping or abrading the rostral region on enclosure walls is associated with captivity stress and interaction with transparent boundaries; could be related with the inability to express a behaviour for which motivation exists such as exploration, thermoregulation, foraging. |
| Decreased physical activity and/or appetite | normal behaviour = inadequate | Decreased physical activity and appetite compared to normal levels are associated with hypothermia, pathology, discomfort/pain, weakness |
| Exhibits aggressive behaviour towards humans (e.g. tries to bite) | normal behaviour = inadequate | Aggressive behaviour toward humans in reptiles can be defensive, and thus associated with fear. |

| | | |
|---|---|---|
| Fast open-mouth breathing with extended neck | normal behaviour = inadequate | Fast open-mouth breathing and gasping for air with extended neck are associated with dyspnoea or hyperthermia |
| The reptile urinates/defecates or regurgitates when in contact or presence of human | normal behaviour = inadequate | Cloacal discharges and regurgitation are defensive behaviours of reptiles and are triggered by fear or stress responses. |
| Puffing-up | not selected for inferential analysis | Puffing up is a defensive display used to increase apparent size in the presence of predators or threats. It is likely linked to stress responses or fear. |
| The reptile moves to a dark place in the enclosure or refuge | not selected for inferential analysis | Ambiguous. Could be related to both positive and negative states, for example brumation, cryptic behaviour, and thermoregulation. |
| Change in colour | not selected for inferential analysis | Ambiguous. Change in skin colour can be linked to defensive displays or to social cues, but also thermoregulation, social behaviour, disease, etc. |
| **Chelonians - section 2 - husbandry** | | |
| **Survey question** | **Variable** | **Justification** |
| Environment (indoor, outdoor, both) | not selected for inferential analysis | Insufficient information to assess adequacy. All lizards were kept in indoor or in mixed scenarios. |
| Enclosure type (terrarium, aquarium, cage, free-roaming, other) | not selected for inferential analysis | Insufficient information to assess adequacy. Whether the enclosure type meets the needs of the reptile depends on many factors such as species, enclosure design, animal size, heat and light sources, etc. |

| | | |
|---|---|---|
| Enclosure dimension (length x width x height) | not selected for inferential analysis | Insufficient information to assess adequacy. Adequacy of enclosure dimension is highly dependent on specimen size, species, enclosure design, and other husbandry variables. |
| Co-habitants | not selected for inferential analysis | All chelonians were housed alone or with up to 3 specimens of the same species. As most are solitary species but tolerate conspecifics, this variable was not predicted to be a relevant for inferential analysis |
| Enclosure walls (transparent, opaque, mixed) | not selected for inferential analysis | Opaque enclosure walls could reduce stress by acting as visual barriers against humans and other aversive stimuli; transparent walls are considered positive enrichment in species that exhibit vigilance behaviour such as cats; it stands logical that mixed set-ups with opaque and transparent sections could allow the best of both options by enabling behavioural self-regulation and choice. Totally transparent enclosure walls were considered inadequate, mixed and opaque walls were considered adequate. Nevertheless, due to the variability in species responses to human presence and dependence on enclosure size and setup, there was insufficient information to assess adequacy. |
| Access to unfiltered UVB light (sunlight or lamp) | no access = inadequate; access = adequate | For omnivorous and herbivorous chelonians UV or direct sunlight are crucial for survival and welfare. Failure to provide this resource results in MBD, a condition accompanied by pain, muscle weakness, fractures, etc. |
| Hours of darkness | not selected for inferential analysis | All reptiles had access to hours of darkness. |
| Enclosure equipment (heat source, heat lamp, UVB lamp, unknown lamp, lamp, other) | not selected for inferential analysis | Insufficient information to assess adequacy. Depends heavily on species and enclosure set-up. Having no heat source or no light source would be considered inadequate, but is accounted for in variables temperature and UV light. |

| | | |
|---|---|---|
| Type of heat source (mat, lamp, cord, plate, inside vs. outside) | not selected for inferential analysis | Insufficient information to assess adequacy. Depends heavily on species and enclosure set-up. Having no heat source or no light source would be considered inadequate, but is accounted for in variables temperature and UV light. |
| Temperature range (min-max) | High-end temperatures more than 2ºC below or 5ºC or more above than the reference = inadequate; high-end temperature with -2 to +4ºC of the reference = adequate. | The adequacy of the temperature range is dependent on species, population within species (e.g. differences in montane or tropical populations) and size. Nevertheless, indicative values are available for many species based on the environmental conditions in the wild and empirical experience in captive settings. High-end temperatures more than 2ºC below or 5ºC or more higher than the reference were considered inadequate. |
| Refuges or hiding places | absent = inadequate; present= adequate | The absence of places to hide or take refuge was considered inadequate because it impedes the expression of behaviours that have high survival value and probably high internal motivation. |
| Access to dry land or islands | not selected for inferential analysis | Most species require dry land, but all respondents except one had dry land and water for their chelonians. |
| Substrate (swimming water, drinking water, moss, bark, wood shavings, clay, coconut fibre, kitchen paper, etc...) | not selected for inferential analysis | Several substrates are used for keeping chelonians and their adequacy is context-dependent. It was not possible to create a uniform criterion for all species. |
| Frequency of enclosure hygiene | not selected for inferential analysis | context dependent, descriptive analysis only |
| Feeding (fruit, insects, shrimp, etc...) | Ca and vit. D provided in some | Considered in combination. Relevant for lizards in captivity. Inadequate: |

| | | |
|---|---|---|
| What kind, frequency and method is used for supplementation (vit D3, Ca, multivit, in formulated feed, dusted, mixed with food, etc…) | form = adequate; not provided = inadequate | absence of dietary source of vitamin D3 and Ca. |

**Turtles - section 3 - bond**

| **Survey question** | **Variable** | **Justification** |
|---|---|---|
| What better defines your lizard (family-member, friend, pet, burden, other) | family member = 1 vs. other bond = 0 | For use as a dependant variable, bond was transformed into a binomial variable to allow logistic regression analysis. Viewing the reptile as a family member was considered the closest bond. |
| How often do you speak to your reptile per week? (never, 1, 2, 3, 4, 5, >5 time) | categorical: never, 1-5 times per week, daily | The responses were collapsed into 3 categories to maintain degrees of freedom manageable. |
| How often do you pet your reptile per week? (never, 1, 2, 3, 4, 5, >5 time) | categorical: never, 1-5 times per week, daily | The responses were collapsed into 3 categories to maintain degrees of freedom manageable. |
| How often do you handle your reptile per week, e.g. picking it up or putting it in your lap? (never, 1, 2, 3, 4, 5, >5 time) | categorical: never, 1-5 times per week, daily | The responses were collapsed into 3 categories to maintain degrees of freedom manageable. |
| When you approach your reptile, how does it react? (approaches, moves away, lifts head, hides, opens mouth, digs, bites, no reaction) | not selected for inferential analysis | For use as an independent variable, behaviours were grouped as predominantly indicative of positive states (e.g. approaching), negative states (biting, evading, hiding, hissing, puffing up) or indeterminate (no reaction). Many responses were difficult to accurately classify, and therefore this variable was not chosen for inferential analysis. |

| | | |
|---|---|---|
| How would you rate the experience of keeping a reptile pet? (very good, good, bad, very bad, no opinion) | binomial: very good or good vs. other response | Keeping reptiles might be tiresome and scarce in gratifying experiences, resulting in a mostly unilateral relation. This could reflect the level of care. |
| How many times have you taken your pet to the veterinarian since you have it? (1, 2, 3, 4, 5, >5) | continuous variable; not selected for inferential analysis. | Divided the number of times reported by the number of years kept as pet. Might need to consider log transformation due to the leverage of frequent visits in very young pets causing outlier effects. |
| Cause of visits to veterinary practice (routine, illness, other) | binomial: routine vs. illness or never | Reptiles commonly mask signs of illness to avoid predation, and therefore are often in life-threatening condition once they start to present clinical signs. Therefore, routine veterinary care, even if infrequent, is considered necessary to ensure reptile welfare. Further, routine veterinary care could provide a source for information on behaviour, reptile care and husbandry. |
| How would you rate you reptile's welfare (very poor, poor, average, good, very good), considering the following definition: *the animal is free of hunger, thirst, discomfort, pain, lesion, illness and fear, and is able to express its normal behaviour.* | binomial: good or very good vs. average or less. | Based on the distribution of the answers, the variable was coded as a binomial variable. |
| **Turtles - section 4 - behaviour** | | |
| **Survey question** | **Variable** | **Justification** |
| The reptile investigates the environment, approaching and smelling objects and/or people | normal behaviour = adequate | Was considered to be related to the expression of exploratory and foraging behaviour. |

| | | |
|---|---|---|
| The reptile exposes itself to light (sunlight or lamp) or approaches the heat source | normal behaviour = adequate | Thermoregulatory behaviour of ectotherms; inability to express this behaviour is likely to be associated with poor welfare. |
| The reptile frequently bumps its head on the glass or enclosure wall or tries to climb the enclosure wall | normal behaviour = inadequate | Head-bumping or abrading the rostral region on enclosure walls is associated with captivity stress and interaction with transparent boundaries; could be related with the inability to express a behaviour for which motivation exists such as exploration, thermoregulation, foraging. |
| Decreased physical activity and/or appetite | normal behaviour = inadequate | Decreased physical activity and appetite compared to normal levels are associated with hypothermia, pathology, discomfort/pain, weakness |
| Exhibits aggressive behaviour towards humans (e.g. tries to bite) | normal behaviour = inadequate | Aggressive behaviour toward humans in reptiles can be defensive, and thus associated with fear. |
| Fast open-mouth breathing with extended neck | normal behaviour = inadequate | Fast open-mouth breathing and gasping for air with extended neck are associated with dyspnoea or hyperthermia |
| The reptile urinates/defecates or regurgitates when in contact or presence of human | normal behaviour = inadequate | Cloacal discharges and regurgitation are defensive behaviours of reptiles and are triggered by fear or stress responses. |
| Retracts into shell in response to human presence or handling | not selected for inferential analysis | Retracting into the shell is a defensive behaviour. When exhibited in response to human presence it is likely linked to stress responses or fear and negative affective states. |
| The reptile moves to a dark place in the enclosure or refuge | not selected for inferential analysis | Ambiguous. Could be related to both positive and negative states, for example brumation, cryptic behaviour, and thermoregulation. |

**B.** List of variables from each questionnaire section (1 to 5) used to build logistic and proportional odds models.

| List of variables used for model building | |
|---|---|
| ***Section 1 - reptile*** | ***Section 4 - behaviour*** |
| Taxonomic group (snake, lizard, chelonian) | Exploration |
| Reptile source (pet shop, breeder, gift or rescue) | Basking |
| ***Section 2 - husbandry*** | Interaction with boundaries |
| Access to light | Anorexia/lethargy |
| Temperature | Human-directed aggression |
| Feeding | Open-mouth breathing |
| Refuge | Defensive discharge |
| Husbandry provision score (0 to 4) | Behavioural knowledge score (0 to 7) |
| ***Section 3 - bond*** | ***Section 5 - respondent demographics*** |
| Bond-type | Age |
| Self-reported welfare | Gender |
| Access to routine veterinary care | Education level |
| | Environment (urban, rural) |
| | Type (breeder, pet-owner) |
